# Free radical scavenging activity and lipoxygenase inhibition of *Mahonia aquifolium *extract and isoquinoline alkaloids

**DOI:** 10.1186/1476-9255-4-15

**Published:** 2007-07-16

**Authors:** Lucia Rackova, Marek Oblozinsky, Daniela Kostalova, Viktor Kettmann, Lydia Bezakova

**Affiliations:** 1 Institute of Experimental Pharmacology, Slovak Academy of Sciences, Dúbravská cesta 9, SK-841 04, Bratislava, Slovakia; 2 Department of Cell and Molecular Biology of Drugs, Faculty of Pharmacy, Comenius University, Odbojárov 10, SK-83232, Bratislava, Slovakia; 3 Department of Pharmacognosy and Botany, Faculty of Pharmacy, Comenius University, Odbojárov 10, SK-83232, Bratislava, Slovakia; 4 Department of Pharmaceutical Analysis and Nuclear Pharmacy, Faculty of Pharmacy, Comenius University, Odbojárov 10, SK-83232, Bratislava, Slovakia

## Abstract

Roots and stem-bark of *Mahonia aquifolium *(Oregon grape) (*Berberidaceae*) are effectively used in the treatment of skin inflammatory conditions.

In the present study, the effect of *Mahonia aquifolium *crude extract and its two representative alkaloid fractions containing protoberberine and bisbenzylisoquinoline (BBIQ) alkaloids on activity of 12-lipoxygenase (12-LOX), was studied. The reactivity with 1,1-diphenyl-2-picryl-hydrazyl (DPPH), a free stable radical, was evaluated to elucidate the rate of possible lipid-derived radical scavenging in the mechanism of the enzyme inhibition.

The results indicate that although the direct radical scavenging mechanism cannot be ruled out in the lipoxygenase inhibition by *Mahonia aquifolium *and its constituents, other mechanisms based on specific interaction between enzyme and alkaloids could play the critical role in the lipoxygenase inhibition rather than non-specific reactivity with free radicals.

## Background

*Mahonia *root and stem bark have long been considered to have anti-inflammatory, anti-bacterial, anti-fungal activity and they are used particularly for treatment of skin diseases [1-4]. They are indicated for treatment of the eczema, psoriasis, and other skin conditions [5].

Alkaloids representing the main compounds in *Mahonia aquifolium*, belong to two major classes: the protoberberines and the bisbenzylisoquinolines (BBIQ). Through bioassay-guided fractionation, protoberberine alkaloids, such as berberine and jatrorrhizine, were isolated as the main active alkaloids responsible for the relevant effects in numerous studies conducted so far [6-8]. In particular, the berberine was reported to exhibit a range of pharmacological and biological activities, and interest has been focused on its antioxidative potential. Berberine was found to inhibit the single-strand cleveage of DNA [9]. It did exhibit a strong superoxide anion radical quenching ability rather than a strong hydroxyl radical scavenging activity [9]. It was reported to exert a protective effect against ONOO^-^, NO^.^, and O_2 _^.-^, induced oxidative damage *in vitro *and to increase cell viability [10].

BBIQ alkaloids, such as berbamine, oxyacanthine, tetrandrine, have been reported to inhibit platelet activation, histamine release, superoxide generation by polymorphonuclear leucocytes (PMNL), lipid peroxidation in some bio-membranes and suppress selectively receptor-mediated phospholipase A_2 _activation, leading to the production of chemical mediators such as prostanoids and leukotrienes, arachidonic acid metabolites [11,12].

Lipoxygenases (LOXs) comprise a family of non-heme iron-containing dioxygenases, representing the key enzymes in the biosynthesis of leukotrienes that have been postulated to play an important role in the pathophysiology of several inflammatory and allergic diseases. The products of LOXs catalysed oxygenation (hydroperoxyeicosatetraenoic acids (HPETE), hydroxyeicosatetraenoic acids (HETE), leukotrienes and lipoxins) apparently are involved in the development of rheumatoid arthritis, psoriasis, asthmatic responses and glomerular nephritis [13].

In our study, the *Mahonia *crude stem bark extract and its fractions identified as tertiary phenolic BBIQ alkaloids (fraction I) and quaternary protoberberine alkaloids (fraction II) were assessed for their effect on the activity of 12-lipoxygenase (12-LOX), isolated from rat lung cytosolic fraction. Since the mechanism of the enzyme inhibition may include reduction of lipidperoxy- or lipidoxy-radicals, the effects of the samples tested on the 12-LOX activity were compared with their abilities to scavenge 1,1-diphenyl-2-picryl-hydrazyl (DPPH), a free stable radical. Two alkaloids (berberine and jatrorrhizine) isolated from fraction II were used as the standards in the *in vitro *assays.

## Methods

### Chemicals

α,α'-diphenyl-β-picrylhydrazyl (DPPH) radicals were obtained from Sigma Chemical Co. (St Louis, MO, USA). Linoleic acid (99%, Sigma) was used as a substrate prepared in solubilized state as described [14]. Other chemicals were purchased from local commercial sources and they were of analytical grade quality.

### Preparation and analysis of Mahoniae stem-bark extract

#### Plant material

The stem-bark of cultivated *Mahonia aquifolium *was collected in October 2002 from Arboretum Mlynany, Faculty of Pharmacy, Bratislava. The authentic specimen is deposited in the herbarium at the Department of Pharmacognosy and Botany, Faculty of Pharmacy (No. Ma 108/9).

### Extract preparation

*Mahonia *stem-bark (*Mahonia aquifolium (Pursh) Nutt*.) was finely powdered and macerated with 62% ethanol (1:10, w/v) for 4 days at room temperature. After removal of the insoluble matter by filtration, the filtrate was concentrated under reduced pressure to yield a dark colored residue. The residue was then dissolved in 10% HCl and the yellow precipitate was filtered through filter paper, the filtrate being concentrated under reduced pressure (filtrate: water = 20:1, w/w). The remaining aqueous extract was crude *Mahonia *extract.

### Fractionation of stem-bark extract Mahoniae

A solution of 25 % NH_4_OH was added to the remaining acidified extract so as to adjust its acidity to pH 8–10 and then it was extracted with diethyl ether (4 × 500 ml). The combined ether extract was evaporated up to dryness under reduced pressure and monitored by thin-layer chromatography so as to yield 6.80 g fraction I of ether- soluble BBIQ alkaloids fraction. The remaining aqueous extract was then acidified to pH 4–5 with 10% HCl and KI solution was added so that a precipitate of quaternary protoberberine alkaloids was obtained. The yellow coloured precipitate was extracted with CHCl_3 _(5 × 1000 ml). The combined chloroform extract was evaporated up to dryness and monitored by thin – layer chromatography, yielding in 19.9 g of chloroform-soluble quaternary protoberberine alkaloid iodide fraction (fraction II).

The yields of fraction I and II (concentrated under reduced pressure and then lyophilized to yield a residue) were 8.6 % and 36.8% w/w, respectively.

The filtrate of crude *Mahonia *extract was concentrated and then lyophilized to yield a residue. The extract yield was 25.6% of the original material weight.

### Isolation of alkaloids from fraction II

Part of the quaternary protoberberine iodides (fraction II) was adsorbed on to silica gel and subjected to a silica gel column chromatography, eluted with chloroform, with the mixtures of chloroform – MeOH (2:1, 1:1. 1:2, 1:4) and MeOH, each about 200 ml with 6 fractions as a result. Fractions 2 and 3 were repeatedly chromatographed on the silica gel column and further purified by recrystalization from EtOH to yield the berberine and jatrorrhizine as iodides.

### Structural analysis of isolated alkaloids

The isolated compounds were identified by direct comparison to the corresponding authentic samples [15]. The purity (>95%) of the above compounds was checked by HPLC analysis [16]. The amount of each alkaloid in the particular fractions was as follows, phenolic BBIQ alkaloids (fraction I): baluchistine 0.074% %, aquifoline 0.071%, oxyacanthine 0.048%, berbamine 0.042%, obamegine 0.038%, aromoline 0.005%, protoberberine alkaloids (fraction II): jatrorrhizine 0.146%, berberine 0.112%, palmatine 0.088%, columbamine 0.030% (w/w).

### In vitro experiments

#### 12-lipoxygenase inhibition assay

A cytosolic fraction from rat (Wistar, male 180 g) lungs representing a source of 12-lipoxygenase was isolated according to the procedure of Kulkarni et al. [17]. The experiments with animals were performed in the laboratory complying Good Laboratory Practice certificate and according to the Law of the National Council of Slovak Republic on the protection of animals No. 115/1995, Edict of Ministry of Agriculture on the breeding of society animals, wild animals and dangerous animals and the protection of laboratory animals No 231/1998 and Council Directive 86/609/EEC on the approximation of laws, regulations and administrative provisions of the Member States regarding the protection of animals used for experimental and other scientific purposes

A spectrophotometric assay for determination of LOX activity was used, i.e. the reaction medium (2.0 ml final volume) contained 50 mM Tris-HCl buffer (pH 8.5), 100 mg of enzyme protein and a solution of linoleic acid prepared in solubilised state [14]. The enzyme inhibitory effect was tested by adding different volumes of the stock solution (0.5 mg/ml) of the extract or fractions tested to the incubation mixture. The pure compounds were tested in final concentrations 15.10^6 ^– 25.10^-6 ^mol/l. The LOX activity was monitored as an increase of the absorbance at 234 nm what reflects the formation of hydroperoxylinoleic acid. The extinction coefficient of 25 mM^-1^. cm^-1 ^was used for calculation of enzyme activity. The inhibitory effect of compounds tested was expressed as IC_50 _and percentage of enzyme activity inhibition.

#### Radical scavenging assay

The free radical scavenging capacity of the compounds tested, crude extract and fractions was determined by using DPPH assay [18-20]. The stock solutions of the samples were prepared in ethanol (0.8 mg/ml for the fractions; 0.47 mg/ml for the standard compounds) and in water (0.7 mg/ml) as far as the crude extract was concerned.

A total 100 μl sample was mixed with a 2900 μl ethanol solution of DPPH (final concentration 60 μM), and the reaction proceeded for 5 hours. After the reaction time, the absorbance change was measured at 515 nm by Hewlett-Packard Diode Array Spectrophotometer 8452A spectrophotometer. Measurements were performed at least triplicate. The standard curves for the reaction of Trolox with DPPH and the readings were then used for calculation of the total antioxidant capacity (TAC) of the sample tested, expressed in μg Trolox equivalents/g [21].

### Statistical analysis

Each experiment was performed in triplicate. Results are expressed as the means ± S.D. Statistical analysis was performed using unpaired Student's t-test using X-Plot v. 2.81 and statistical significance is expressed as *, p < 0.05.

## Results

### Lipoxygenase inhibitory effect

The LOX activity (12-arachidonate LOX purified from rat lung cytosol fraction) was monitored as an increase in the absorbance at 234 nm, which reflects the formation of hydroperoxylinoleic acid. The highest inhibitory effect was obtained for crude extract of *Mahonia aquifolium *(IC_50 _= 0.76.10^-3 ^± 0.12.10^-3 ^g/L) (Table 1). As far as the two alkaloid fractions were compared, a higher inhibition of LOX is caused by fraction I (IC_50 _= 4.63.10^-3 ^± 1.03.10^-3 ^g/L) than by fraction II (IC_50 _= 6.10.10^-3 ^± 0.98.10^-3 ^g/L). IC_50 _values, determined for the isolated compounds, were 30.5.10^-6 ^± 2.87.10^-6 ^mol/l (berberine) and 17.5.10^-6 ^± 1.27.10^-6 ^mol/l (jatrorrhizine) (Table 2).

**Table 1:** Results of lipoxygenase inhibitory effects of crude *Mahonia *extract and two representative alkaloid fractions tested.

	Final concentration (g/L)	Activity of lipoxygenase (kat)	% Inhibition	IC_50 _(g/L)
Crude *Mahonia *extract	0.50.10^-3^	7.40 ± 0.89	43.03 ± 0.54	0.76.10^-3 ^± 0.12.10^-3^
	0.75.10^-3^	6.34 ± 7.63	51.19 ± 0.96*	
	1.25.10^-3^	5.2 8 ± 0.78	59.32 ± 1.39*	
	1.88.10^-3^	3.53 ± 0.34	72.80 ± 1.54*	
	2.50.10^-3^	2.92 ± 0.28	78.80 ± 1.72*	
Control	-	12.99 ± 1.23	-	
Fraction I				
	1.25.10^-3^	1.16 ± 0.11	16.54 ± 7.67	4.63.10^-3 ^± 1.03.10^-3^
	2.50.10^-3^	0.94 ± 0.09	32.23 ± 2.63	
	3.75.10^-3^	0.71 ± 0.09	47.33 ± 2.24	
	5.10^-3^	0.63 ± 0.07	54.67 ± 1.64*	
	6.25.10^-3^	0.55 ± 0.06	60.43 ± 2.36*	
	7.50.10^-3^	0.34 ± 0.04	75.39 ± 2.10*	
Control	-	1.39 ± 0.14	-	
Fraction II				
	1.25.10^-3^	1.24 ± 0.12	10.93 ± 4.76	6.10.10^-3 ^± 0.98.10^-3^
	2.50.10^-3^	1.11 ± 0.10	18.27 ± 6.23	
	3.75.10^-3^	0.95 ± 0.09	31.08 ± 2.25	
	5.10^-3^	0.87 ± 0.09	39.28 ± 2.47	
	6.25.10^-3^	0.64 ± 0.07	53.67 ± 2.33*	
	7.50.10^-3^	0.56 ± 0.06	60.86 ± 3.04*	
Control	-	1.39 ± 0.24	-	

Lipoxygenase activity was determined as absorbance increase at λ_max _= 234 nm at 3 minutes of incubation with or without inhibitor tested. Values of hydroperoxide content and lipoxygenase activity were calculated from equation c = A.V/ε.l.v, where A is the value of absorbance increase, V is the volume of incubation mixture, ε is the extinction coefficient for linoleic acid (25.10^-3 ^mol.l.cm^-1^), l is the length of the cuvette (1 cm) and v is the volume of enzyme (0.015 ml). Results are presented as percent of control ± SD, n = 3, * p < 0.05 vs. controls

**Table 2:** Results of lipoxygenase inhibitory effects of two representative alkaloids isolated from *Mahonia aq*. crude extract.

	Final concentration (mol/l)	Activity of lipoxygenase	% Inhibition	IC_50 _(mol/L)
Aqueous solution of jatrorrhizine (0.01M)				
	15.10^-6^	118.71 ± 4.37	22.05 ± 1.37	17.50.10^-6 ^± 1.27.10^-6^
	20.10^-6^	23.89 ± 1.83	84.32 ± 3.74*	
	25.10^-6^	12.73 ± 1.24	91.58 ± 2.91*	
Control	-	152.29 ± 5.37	-	
Aqueous solution of berberine (0.01M)				
	15.10^-6^	233.19 ± 7.41	14.88 ± 0.97	30.50.10^-6 ^± 2.87.10^-6^
	20.10^-6^	213.40 ± 6.93	22.11± 1.24	
	25.10^-6^	157.02 ± 4.21	42.69 ± 2.38	
Control	-	273.96 ± 8.21	-	

Lipoxygenase activity was determined as absorbance increase at λ_max _= 234 nm at 3 minutes of incubation with or without inhibitor tested. Values of hydroperoxide content and lipoxygenase activity were calculated from equation c = A.V/ε.l.v, where A is the value of absorbance increase, V is the volume of incubation mixture, ε is the extinction coefficient for linolic acid (25.10^-3 ^mol.l.cm^-1^), l is the length of the cuvette (1 cm) and v is the volume of enzyme (0.015 ml). Results are presented as percent of control ± SD, n = 3, * p < 0.05 vs. controls

### Radical scavenging activity

In order to assess the radical scavenging potential of the crude extract, fractions I, II and the compounds isolated, the reactivity towards the stable free radical DPPH was measured at 518 nm by measurement of absorbance decrease of the reaction mixture after 5 hour-reaction time. The results expressed as TAC in μg Trolox equivalents/g (Table 3) [21] showed that the most potent antiradical reactivity may be attributed to the phenolic BBIQ alkaloids constituting fraction I, whereas a remarkably lower antioxidant capacity was obtained for the samples of crude extract, fraction II and berberine with comparable values of TAC. Jatrorrhizine, the phenolic alkaloid, showed a significantly higher antiradical activity than berberine, the other standard used.

**Table 3:** TAC of *Mahonia *crude extract, fraction Iand II and two representative alkaloids.

	TAC, μg Trolox equiv/g
Fraction I	209 833.3 ± 4880.0
Fraction II	39 969.7 ± 3007.3
*Mahonia *crude extract	71 406.8 ± 5822.6
Jatrorrhizine	116 519.5 ± 1601.4
Berberine	38 441.6 ± 3171.3

A total 100 μl sample was mixed with a 2900 μl ethanol solution of DPPH (final concentration 60 μM), and the reaction continued for 5 hours. The final concentrations in the reaction mixture of the samples tested were as follows: 0.027 mg/ml for the fractions; 0.016 mg/ml for the standard compounds and 0.023 mg/ml for crude extract in water. Total antioxidant capacity (TAC) was calculated using standard curves for the reaction of Trolox with DPPH and the absorbance change at 515 nm after the reaction time, and the readings were then used to calculate TAC in the sample, expressed in μg Trolox equivalents/g. Each result is expressed as mean ± S.D. for three values.

## Discussion

This study elucidates the possible contribution of the radical scavenging effect to the lipoxygenase inhibitory mechanism of the crude *Mahonia aquifolium *extract and two isolated alkaloid fractions, containing phenolic BBIQ alkaloids (fraction I) and protoberberine alkaloids (fraction II). Two representative alkaloids, jatrorrhizine, possessing phenolic moiety and its non-hydroxylated analogue, berberine, were isolated from fraction II and used in the assays of the antiradical and anti-lipoxygenase activity as standards.

LOXs are the family of the key enzyme in the biosynthesis of leukotrienes that are postulated to play an important role in the pathophysiology of several inflammatory disseases. According to the currently used nomenclature, the LOXs are classified with respect to their positional specificity of arachidonic acid oxygenation (5-LOX, 9-LOX, 12-LOX, 15-LOX) [22].

In the present work, the LOX inhibitory properties were tested on purified arachidonate-12-LOX from rat lung cytosol fraction. 12-LOX metabolites of arachidonic acid are potent mediators of inflammation and one of the regulators of pulmonary vascular tone, and their production is increased during lungs vascular injury [23]. 12-lipoxygenase products (12-HETE) are also potent mediators of cutaneous inflammation. These eicosanoids have been found in biologically active amounts in scales and samples of lesional psoriatic skin [1].

LOX are sensitive to antioxidants, and the most common way of their action may consist in inhibition of lipid hydroperoxide formation due to scavenging of lipidoxy- or lipidperoxy-radicals formed in course of enzymic peroxidation. This can limit the availability of lipid hydroperoxide substrate necessary for the catalytic cycle of LOX.

An inhibition of the lipoxygenases by antioxidants can be also attained via chelation of its non-heme bound iron [24] or by reduction of its ferric form [25-27], suggesting a competitive kind of inhibition. However, non-competititve or mixed competitive/non-competitive inhibition of LOX was also shown for tocopherol acetate or β-carotene, respectively [28].

In our previous studies, an LOX inhibition potential was obtained for series of the BBIQ alkaloids [16], as well as the protoberberine and aporphine alkaloids [29] and the values of enzyme inhibition showed a strong linear correlation with the antiperoxidant effects of the compounds tested. These results indicated that the mechanism of the LOX inhibition effect could be partly explained by direct reduction of peroxy- and alkoxy-radicals.

However, a simpler assay system is required to elucidate the role of a direct free radical scavenging in the lipoxygenase inhibition by *Mahonia aquifolium* alkaloids, since in the previously used liposomal model of lipid peroxidation (induced by the Fenton system), the resulting effect may be considered as a superimposition of reactivity of the compounds with free radicals, chelation of Fe^2+ ^or partition process in the heterogeneous membrane system. For this reasons, we evaluated the consistency of anti-lipoxygenase activity of the samples tested with their reactivity with DPPH, a free radical model. DPPH is also considered as a good kinetic model for peroxyl radicals [18-20].

In order to put the common parameter for comparison of the antiradical activity of the fractions and the crude extract with the pure compounds, we have evaluated the total antioxidant activity (TAC) [21] of the samples tested. In consistency with our previous results [30], the highest value of TAC was obtained for fraction I containing bisbenzylisoquinoline (BBIQ) alkaloids (baluchistine, aquifoline oxyacanthine, berbamine, obamegine, aromoline), possessing properties of phenolic antioxidants (Table 3). The crude extract and fraction II (containing protoberberine alkaloids with reduced number of free OH moieties) showed a remarkably lower and comparable antiradical activity. Contrary to the previously obtained results for lipid peroxidation and lipoxygenase inhibition assay [17,29], we obtained a remarkably low antiradical activity for the *Mahonia *crude extract.

As shown in Table 1, the strongest lipoxygenase inhibition was obtained for the crude extract (IC_50 _= 0.76.10^-3 ^± 0.12.10^-3^g/L). Although, analogously to TAC values, phenolic BBIQ fraction I showed the stronger LOX inhibition effect (IC_50 _= 4.63 ± 1.03.10^-3^gL) than protoberberine alkaloid fraction II (IC_50 _= 6.1.10^-3 ^± 0.98.10^-3^g/L), the difference between their enzyme inhibition activities was not so remarkable as that between their antiradical activities.

These results suggest that a scavenging effect may not be a critical factor behind the inhibition of LOX by the crude Mahonia extract and its two fractions and the inhibitory effects are possibly due to specific interaction of the constituents with the enzyme. As mentioned above, an interaction with iron atom at the enzyme catalytic centre may be involved in the LOX inhibition mechanism. Regarding catecholic type of structures of *Mahonia aquifolium* constituents, the LOX activity could be abolished via formation of stable chelates of its non-heme iron, as it has been shown for aporphine alkaloid, apomorphine [31], suggesting thus a competitive mode of enzyme inhibition. This could also explain the good correlation between LOX inhibition effects of the Mahonia extract and its constituents and their protective effects against lipid peroxidation [17,29], exerted probably via interaction with Fe^2+ ^of the initiator system (FeSO_4_/H_2_O_2_). However, regarding the size of BBIQ molecules, other types of inhibition involving preferential interaction with other than enzyme's catalytic cavity (i.e. allosteric, uncompetitive or non-competitive inhibition), cannot be excluded.

Expectably, a hydroxylated alkaloid jatrorrhizine showed three times higher antiradical reactivity expressed as TAC than its non-hydroxylated analogue, berberine (Table 3). The radical scavenging properties related to OH moiety on the alkaloid skeleton of jatrorrhizine may be also responsible for the increase of enzyme inhibitory effect (IC_50 _= 17.5.10^-6 ^± 1.27.10^-6 ^mol/L, Table 2) in comparison to berberine (IC_50 _= 30.5.10^-6 ^± 2.87.10^-6 ^mol/L), as indicated by the consistency with results of DPPH radical scavenging assay.

In conclusion, our results indicate that although the direct scavenging of free radicals cannot be ruled out in the mechanism of lipoxygenase inhibition by the *Mahonia aquifolium *extract and its two representative fractions containing BBIQ and protoberberine alkaloids, this does not seem to be the critical mechanism through which *Mahonia aquifolium* and its constituents exert their inhibition effects and these can be rather explained by a direct specific interaction of the constituents with LOX enzyme.

## Competing interests

The author(s) declare that they have no competing interests.

## Authors' contributions

L.R., L.B. and M.O. carried out all of the in vitro experiments reported in this manuscript. L.B. and V.K. participated in design of the study. D.K. carried out the methods of extract and fractions isolation. All authors read and approved the final manuscript.
